# Supplementary material for: Indoor mobility challenges among older adults: A systematic review of barriers and limitations
Source: PLoS One. 2025 Jun 4;20(6):e0325064. doi: 10.1371/journal.pone.0325064 (PMC12136408; doi:10.1371/journal.pone.0325064)
Supplement: S1 File — (DOCX) [file pone.0325064.s001.docx]

**S1 File: Search strings used in the current study**

The following search string was used in PubMed and was adapted to Embase, CINAHL Plus, and PsycINFO

1. Mobility
2. Motion
3. Movement
4. Life space
5. #1 OR #2 OR #3 OR #4
6. indoor
7. inside
8. internal
9. home
10. domiciliary
11. residential
12. #6 OR #7 OR #8 OR #9 OR #10 OR #11
13. older adult
14. geriatric
15. senior
16. ageing
17. elder*
18. old*
19. age*
20. retired person
21. pensioner
22. old-timer
23. #13 OR #14 OR #15 OR #16 OR #17 OR #18 OR #19 OR #20 OR #21 OR #22
24. challenge
25. problem
26. difficulty
27. limitation
28. #24 OR #25 OR #26 OR #27
29. #5 AND #12
30. #29 AND #23
31. #30 AND #28
