# Supplementary material for: Indoor mobility challenges among older adults: A systematic review of barriers and limitations
Source: PLoS One. 2025 Jun 4;20(6):e0325064. doi: 10.1371/journal.pone.0325064 (PMC12136408; doi:10.1371/journal.pone.0325064)
Supplement: S2 File — (DOCX) [file pone.0325064.s002.docx]

**S2 File:** **Data extraction form for the systematic review**

|  | Research Review Questions | Response |
| --- | --- | --- |
| **Study Attributes** | | |
| 1. | First Author | ---------------------- |
| 2. | Year of Publication | ---------------------- |
| 3. | Title of Publication | ---------------------- |
| 4. | Study Design | ---------------------- |
| **Study Population** | | |
| 5. | Sample Size | ---------------------- |
| 6. | Mean Age | ---------------------- |
| 7. | % of females | ---------------------- |
| 8. | Setting | ---------------------- |
| 9. | Weight (kg) | ---------------------- |
| 10. | BMI (kg/m2) | ---------------------- |
| 11. | Muscle mass (%) | ---------------------- |
| 12 | Comorbitities | ---------------------- |
| 13 | Mean no of medications | ---------------------- |
| **Mobility assessment** | | |
| 14 | Mean steps /day (SD) | ---------------------- |
| 15 | Activities | Mild activity  Moderate activity |
| a | Mean ADL score |  |
| b | Mean IADL score |  |
| c. | Life space mobility score | ---------------------- |
| 16 | Physical assessment | ---------------------- |
| a | Maximal leg power | ---------------------- |
| b | Optimal velocity | ---------------------- |
| c | Optimal torque | ---------------------- |
| 17 | Walking speed |  |
| a | 6m test | ---------------------- |
| b | 5m chair rise | ---------------------- |
| c | 5m stair climb | ---------------------- |
| 16 | Cognitive state | ---------------------- |
| 17 | Quality of life |  |
| 18 | Tilburg Frailty Indicator | ----------------------------- |
| **Reviewer details** | | |
| 19. | Data extraction 1 and date | ----------------------------- |
| 20. | Data extraction 2 and date | ----------------------------- |
| 21 | Reviewed and adjudicated by | ----------------------------- |

The full data extraction table can be accessed here

<https://docs.google.com/spreadsheets/d/1njMFFDKn9vgsnEZbZy1pTWJqXlWfw2BVvfA0awAPUPg/edit?usp=sharing>
