# Supplementary material for: Indoor mobility challenges among older adults: A systematic review of barriers and limitations
Source: PLoS One. 2025 Jun 4;20(6):e0325064. doi: 10.1371/journal.pone.0325064 (PMC12136408; doi:10.1371/journal.pone.0325064)
Supplement: S1 Table — (DOCX) [file pone.0325064.s004.docx]

**S1 Table: List of excluded studies with reasons**

| **No.** | **Reference** | **Reasons for exclusion** |
| --- | --- | --- |
| 1 | Johannesen A, Petersen J, Avlund K. Satisfaction in everyday life for frail 85-year-old adults: A Danish population study. Scand J Occup Ther. 2004;11:3-11. doi:10.1080/11038120410019045 | Study is assessing everyday life satisfaction for frail older adults instead of indoor mobility challenges. |
| 2 | Levasseur M, Filiatrault J, Larivière N, Trépanier J, Lévesque MH, Beaudry M, Parisien M, Provencher V, Couturier Y, Champoux N, Corriveau H, Carbonneau H, Sirois F. Influence of Lifestyle Redesign on Health, Social Participation, Leisure, and Mobility of Older French-Canadians. Am J Occup Ther. 2019 Sep/Oct;73(5):7305205030p1–7305205030p18. doi: 10.5014/ajot.2019.031732 | The study aimed to investigate the impact of Lifestyle Redesign instead of indoor mobility challenges faced by older people. |
| 3 | Ekdahl C, Dehlin O, Samuelsson G, Sartor A-C. Changes in physical functional capacity from 71 to 78 years of age for a population living at home. Scand J Caring Sci. 1987;1:33-39. doi:10.1111/j.1471-6712.1987.tb00297.x | The study primarily assessed parameters of outdoor mobility in older people. |
| 4 | Thapa PB, Gideon P, Fought RL, Kormicki M, Ray WA. Comparison of clinical and biomechanical measures of balance and mobility in elderly nursing home residents. J Am Geriatr Soc. 1994;42(5):493-500. doi:10.1111/j.1532-5415.1994.tb04970.x | The study focuses on comparing biomechanical force platform measurements and clinical measures of balance and mobility, without assessing the risks and limitations related to indoor mobility. |
| 5 | Kottner J, Halfens R, Dassen T. Interrater reliability and agreement of the Care Dependency Scale in the home care setting in the Netherlands. Scand J Caring Sci. 2010;24 Suppl 1:56-61. doi:10.1111/j.1471-6712.2009.00765.x | This study focuses on the interrater reliability and agreement of the CDS in the home care setting and does not explore issues related to indoor mobility challenges. |
| 6 | Taylor J, Hill H, Kay K. An integrated practice approach to mobility care for older people. Nurs Stand (RCN). 2016 Jul 20-26;30(29):51-60. doi: 10.7748/ns.30.29.51.s47 | The study does not explore the aspect of indoor mobility challenges among older people. |
| 7 | Blocker WP Jr. Maintaining functional independence by mobilizing the aged. Geriatrics. 1992;47(1): | The study does not delve into the specific challenges older adults might encounter in indoor settings. |
| 8 | Bohannon RW. Measurement of gait speed of older adults is feasible and informative in a home-care setting. J Geriatr Phys Ther. 2009;32(1):22-23. doi:10.1519/00139143-200932010-00005 | The study focuses on the feasibility and informativeness of measuring gait speed in a home care setting but does not investigate the specific indoor mobility challenges among older adults. |
| 9 | Mancinella A, Mancinella M, Marigliano B, Marigliano V. Benessere ed attività fisica nell'anziano fragile [Well-being and physical activity in the frail elderly]. Recenti progressi in medicina. 2013;104(4):163-167 | Study is published in Italian |
| 10 | Virokannas H, Pirinen M, Kirvesoja H, Väyrynen S, Kemppainen ML, Koski K. Safety and indoor mobility in the elderly. Stud Health Technol Inform. 1998;48:398-401 | Study is published in Danish |
