# Supplementary material for: Indoor mobility challenges among older adults: A systematic review of barriers and limitations
Source: PLoS One. 2025 Jun 4;20(6):e0325064. doi: 10.1371/journal.pone.0325064 (PMC12136408; doi:10.1371/journal.pone.0325064)
Supplement: S2 Table — (DOCX) [file pone.0325064.s005.docx]

**S2 Table: Newcastle-Ottawa Scale (NOS) Assessment Tool**

| **Studies** | Selection | Comparability | Outcome | **Total** |
| --- | --- | --- | --- | --- |
| Cress, 2011 | ★★★ |  | ★★ | **5** |
| Brustio, 2022 | ★★★ | ★★ | ★★★ | **8** |
| Martin, 2021 | ★★★ | ★★ | ★★ | **7** |
| Mänty, 2012 | ★★★ | ★ | ★★ | **6** |
| Geren, 2006 | ★★★ | ★ | ★★ | **6** |
| Tsai, 2014 | ★★★ | ★★ | ★★★ | **8** |
| May, 1985 | ★★★ | ★ | ★★★ | **7** |
| Clemencon, 2008 | ★★★ | ★ | ★★★ | **7** |

***Poor: <4 stars***

***Medium: 4-6 stars***

***High: ≥7 stars***
