# Supplementary material for: Indoor mobility challenges among older adults: A systematic review of barriers and limitations
Source: PLoS One. 2025 Jun 4;20(6):e0325064. doi: 10.1371/journal.pone.0325064 (PMC12136408; doi:10.1371/journal.pone.0325064)
Supplement: S3 Table — (DOCX) [file pone.0325064.s006.docx]

**S3 Table. Joanna Briggs Institute (JBI) Assessment Tool**

| **Questions** | **Brim et al., 2021** |
| --- | --- |
| Is there congruity between the stated philosophical perspective and the research methodology? | Yes |
| Is there congruity between the research methodology and the research question or objectives? | Yes |
| Is there congruity between the research methodology and the methods used to collect data? | Yes |
| Is there congruity between the research methodology and the representation and analysis of data? | Yes |
| Is there congruity between the research methodology and the interpretation of results? | Yes |
| Is there a statement locating the researcher culturally or theoretically? | No |
| Is the influence of the researcher on the research, and vice-versa, addressed? | No |
| Are participants, and their voices, adequately represented? | Yes |
| Is the research ethical according to current criteria or, for recent studies,  and is there evidence of ethical approval by an appropriate body? | Unclear |
| Do the conclusions drawn in the research report flow from the analysis, or interpretation, of the data? | Yes |
|  |  |
