# Supplementary material for: Indoor mobility challenges among older adults: A systematic review of barriers and limitations
Source: PLoS One. 2025 Jun 4;20(6):e0325064. doi: 10.1371/journal.pone.0325064 (PMC12136408; doi:10.1371/journal.pone.0325064)
Supplement: Attachment — Submitted filename: PLOS One letter.docx [file pone.0325064.s008.docx]

10 May 2025

Jan Chrusciel

Academic Editor

PLOS ONE

Dear Dr Jan Chrusciel,

Thank you very much for kindly considering our manuscript entitled “Indoor mobility challenges among older adults: A systematic review of barriers and limitations” for submission to PLOS ONE. As requested by the reviewer, we have made substantial changes to our manuscript. These changes are delineated as follow:-

**The editor had the following comment**s

**Comment #1  Please ensure that your manuscript meets PLOS ONE's style requirements, including those for file naming. The PLOS ONE style templates can be found at**

[https://journals.plos.org/plosone/s/file?id=wjVg/PLOSOne_formatting_sample_main_body.pdf](https://track.editorialmanager.com/CL0/https:%2F%2Fjournals.plos.org%2Fplosone%2Fs%2Ffile%3Fid=wjVg%2FPLOSOne_formatting_sample_main_body.pdf/1/010f019568acd758-937b1815-9e86-4145-b263-11853a87d24e-000000/OihNmbF_9nmE8r103lwDNUxH5sEdPVcxJZxH8Xu2iss=201)  and

[https://journals.plos.org/plosone/s/file?id=ba62/PLOSOne_formatting_sample_title_authors_affiliations.pdf](https://track.editorialmanager.com/CL0/https:%2F%2Fjournals.plos.org%2Fplosone%2Fs%2Ffile%3Fid=ba62%2FPLOSOne_formatting_sample_title_authors_affiliations.pdf/1/010f019568acd758-937b1815-9e86-4145-b263-11853a87d24e-000000/OxxCUKcuBPcRILys1rBJacu1gw1xNaPMtaokjsDqXQw=201)

We take note and edited our manuscript to conform with PLOS ONE’s style requirements.

**Comment #2. We note that the grant information you provided in the ‘Funding Information’ and ‘Financial Disclosure’ sections do not match. When you resubmit, please ensure that you provide the correct grant numbers for the awards you received for your study in the ‘Funding Information’ section.**

We take note and edited it to ensure it matches

**Comment #3. As required by our policy on Data Availability, please ensure your manuscript or supplementary information includes the following: A numbered table of all studies identified in the literature search, including those that were excluded from the analyses.**

We take note and edited our supplementary information as required as S1 Table

**Comment #4. For every excluded study, the table should list the reason(s) for exclusion.**

We take note and edited our supplementary information as required as S1 Table.

**Comment #5. If any of the included studies are unpublished, include a link (URL) to the primary source or detailed information about how the content can be accessed.**

We take note and have ensured compliance. All our included articles have been published in journals and therefore can be easily accessed.

**Comment #6. A table of all data extracted from the primary research sources for the systematic review and/or meta-analysis. The table must include the following information for each study:**

- **Name of data extractors and date of data extraction.**
- **Confirmation that the study was eligible to be included in the review.**
- **All data extracted from each study for the reported systematic review and/or meta-analysis that would be needed to replicate your analyses.**
- **If data or supporting information were obtained from another source (e.g. correspondence with the author of the original research article), please provide the source of data and dates on which the data/information were obtained by your research group.**
- **If applicable for your analysis, a table showing the completed risk of bias and quality/certainty assessments for each study or outcome.  Please ensure this is provided for each domain or parameter assessed. For example, if you used the Cochrane risk-of-bias tool for randomized trials, provide answers to each of the signalling questions for each study. If you used GRADE to assess certainty of evidence, provide judgements about each of the quality of evidence factor. This should be provided for each outcome.**
- **An explanation of how missing data were handled.**
- **This information can be included in the main text, supplementary information, or relevant data repository. Please note that providing these underlying data is a requirement for publication in this journal, and if these data are not provided your manuscript might be rejected.**

We take note of these requirements. We have included a copy of our data extraction table used in our study into the supplementary information.

**Reviewer #1 had the following comments.**

**Comment #1. This systematic review presents a well-structured and informative synthesis of indoor mobility challenges among older adults. The study effectively outlines its objectives, methods, and findings, with a clear adherence to PRISMA guidelines and a well-defined search strategy. The thematic synthesis is appropriate, and the risk-of-bias assessment strengthens methodological rigor. However, minor grammatical inconsistencies, structural redundancies, and inconsistencies in terminology impact readability. Below are the key areas for refinement**

We thank the reviewer for the encouraging comments and suggestions. We have edited the manuscript according to the suggestions of the reviewer.

**Comment #2. 1. Title Revision for Clarity & Precision: Suggested: " Indoor Mobility Challenges Among Older Adults: A Systematic Review of Barriers and Limitations."**

We thank the reviewer for the suggestion. We have now edited our manuscript title as suggested to read as follow:-

Indoor Mobility Challenges Among Older Adults: A Systematic Review of Barriers and Limitations

**Comment #3. Revise redundant phrases (e.g., "worsening of health issues due to age-related declines in physical strength and mobility").**

We take note and thank the reviewer for the suggestion. We have revised the abstract introduction as follow:-

The global population is projected to double by 2050 with most older adults expressing preference to age in place. Despite this demographic shift, indoor mobility challenges which directly impact independence, safety and quality of life remains poorly understudied, creating a critical knowledge gap for effective intervention development.

**Comment #4. Improve clarity in methodology description:
"Two reviewers independently conducted data abstraction, risk-of-bias assessment, and quality evaluation."**

We take note and thank the reviewer for the suggestion. We have revised the abstract methods as follow:-

A systematic literature search was conducted on four databases from inception to September 2024. Inclusion criteria included: (1) participants aged 60 years and above; (2) were primary data on self-reported indoor mobility challenges; and (3) published in English language. Two reviewers independently performed data abstraction, evaluated the risk-of-bias and quality of included article using the NewCastle Ottawa Scale for cross-sectional or Joanna Briggs Institute Checklist for qualitative studies.

**Comment #5. Minor grammatical refinements (e.g., "Research have shown" → "Research has shown").**

We thank the reviewer for the suggestion. We have now edited the introduction as suggested and also requested a colleague who is an English native speaker to edit our manuscript for clarity.

Research has shown that grip strength in men and walking speed in women declines with age

**Comment #6 Ensure terminology consistency by using "older adults" uniformly.**

We agree this was unclear. We have checked to ensure consistency across our manuscript as suggested

**Comment #7. Methodology Refinements: Revise: "Data analysis was performed using NVivo 12 (Lumivero)." to "Data were analyzed using NVivo 12 (Lumivero)."**

**We thank the reviewer for the suggestion. We have now edited the methods to state the following:-**

Data were analysed using NVivo 12 (Lumivero).

**Comment #8. Improve consistency in citation formatting and refine descriptions for readability.**

We take note and have edited the citation formatting for readability.

**Comment #9. Results Section Refinements:Sentence restructuring for clarity:
"Mobility limitations negatively impacted daily activities, including feeding, bathing, grooming, dressing, continence (bowel and bladder), toilet use, transfers, general mobility, and stair climbing."**

We thank the reviewer for the suggestion and have edited the sentence as suggested.

**Comment #10. Ensure correct reference formatting (e.g., "Appendix Tables 2 and 3").**

We take note and checked the reference formatting to ensure consistency.

**Comment #11. Improve thematic descriptions for readability and flow.**

We take note of the comment and have now reedited our thematic description for readability and flow based on the comments from both reviewers as follow:-

**Fatigability**

Activity tolerance in older adults encompasses the ability to engage in various activities and maintain different body positions, including walking, climbing stairs, and performing kitchen or cleaning tasks. Older adults commonly attribute difficulties in these activities to self-reported musculoskeletal or cardiopulmonary conditions.[1] Two studies also reported how indoor mobility activities of older adults is limited due to reduced endurance or fatigability, leading to slower walking and gait speeds.[2, 3] Indeed, nearly one in every four older adult (22.9%) were reported to experience fatigue when transferring or moving indoor, with 32.1% reported fatigue especially among those with co-morbidities.[2] In another study by Clemencon and colleagues, the authors that leg muscle mass can be an important parameter to use as it predicts fatigability and therefore physical performance.

As individuals age, the prevalence of chronic diseases rises, with more than half of the population (68.8%) requiring pharmacotherapies.[4] The use of medication was found to impact their mobility as older adults reported experiencing fatigue with these medication use. In particular, there was a 10% increase in the likelihood of reporting indoor mobility-related fatigue with every additional medication taken by an older adult.[2] Additionally, older adults reporting musculoskeletal pain were twice as likely to report fatigue, and the presence of depressive symptoms was significantly associated with reported fatigability, which led to reduced mobility.[2] Among frail older adults, this fatigue led to decreased step counts, increased sedentary behaviour, and greater levels of disability compared to their more robust cohort.[4]

**Poor balance**

Functional mobility and balance, stemming from previous falls, are common health barriers among older adults, leading to decreased balance, unsteadiness, and concerns about night time bathroom accessibility.[15] While home-dwelling elders generally exhibit better balance compared to institutionalised elderly individuals, approximately one-third of older adults expressed fear of falling, significantly impeding their mobility.[17] May and colleagues established an inverse relationship between balance (measured by mean sway path) and mobility index, indicating that better balance corresponds to higher mobility scores. Conversely, individuals with a fear of falling display lower mobility, slower walking speeds, and higher mean sway path values, indicating poorer balance.[19]While reasons for the poor balance vary, these were identified due to several factors, including the use of medications, the consumption of alcohol, inner ear imbalance as well as certain medical conditions, such as cognitive impairment.[19] Importantly, for an older adult to be mobile out of doors, this required them to have a moderate or rapid gait speed, which are often lacking among those with poor balance.[5]

The review also noted an association between cognitive impairment and the likelihood of misjudging daily activities, especially those living in nursing homes.[6] These results underscore the importance of considering cognitive state when evaluating self-assessed mobility, especially for those living in nursing homes.[6] Mobility limitations negatively impacted daily activities including feeding, bathing, grooming, dressing, continence (bowels and bladder), toilet use, transfer, mobility, and stairs climbing.[6] Studies also reported that due to hearing loss, bowel and bladder issues, and vision impairments contribute to poor balance, thereby increasing the risk of falls.[5]

**Home environmental barriers**

The home environment was identified as the key factor that can either support or hinder the indoor mobility of older adults, often contributing to falls due to poor balance. In particular, older adults described the importance of creating a safe and accessible living environment at home to support indoor mobility among older adults.[1] For instance, hazards such as damaged or broken stairs and railings, poor lighting, loose areas of rugs and obstacles in walkways were reported to cause these older adults to be wary, as they worry about the risk of falling.[1] Due to these factors, older adults were reported to limit their movements indoors which led to reduced mobility indoors and decline in independence.[5]

**Reduced strength and limb weakness**

Both reduced physical strength and limb weaknesses due to ageing among older adults were reported to significantly impact the ability of older adults to move indoors, with a concomitant toll on access to daily activities. For instance, studies have reported that older adults experience a reduced range of motion, which limits their mobility, flexibility, and walking speed.[7] These limitations were more pronounced among those living in retirement communities, as they were reported to engage in fewer daily steps, and exhibit lower activity levels[4, 7, 8] compared to those living independently in the community[9]. This limitation was even more pronounced among those with frailty, where Brustio and colleagues reported that among frail older adults, they were more likely to experience indoor mobility restrictions and higher levels of disability.[4]

**Comment #12. The conclusion would benefit from a stronger emphasis on the implications for policy and practice.**

We agree and thank the reviewer for the suggestion. Our revised conclusion now reads as follow

In summary, this study identified challenges older adults face in indoor mobility, including physical limitations, frailty, chronic diseases, medication use, and environmental barriers. To optimize implementation, future studies should examine the cost-effectiveness of different implementation models across diverse geographic and socioeconomic contexts to create more adaptable approaches to aging-in-place. Our findings also challenge the current paradigm that aging-in-place can succeed without systematic support structures. The evidence demands not just acknowledgment of these challenges but decisive action to transform how we support indoor mobility among older adults.

**Comment #13. Revise: "A comprehensive environmental assessments and home modifications can be considered." to: "Comprehensive environmental assessment and home modifications should be considered to support ageing in place."**

We take note and have edited the sentence as suggested.

**Reviewer #2: Thank you for asking me to review this article which is interesting but required further analysis. Below are my comments.**

**Comment #1. The search strategy is not included in the appendix of the study.**

We apologise for the oversight. We have now appended a new appendix which details the search strategy for one of the databases. The search strategy was subsequently adjusted for the other databases.

**Comment #2. The authors use NewCastle Ottawa Scale and Joanna Briggs Institute checklist. Since some included studies were mixed method how was this dealt with? Why a MM checklist not used instead?**

We apologise for the confusion. There were no mixed methods study and all included were quantitative studies which had used different methods of collections including self-administration or interviewer assisted, except for one study which was qualitative in nature. We have now revised our table of characteristics to clarify this and thank the reviewer for pointing this out.

**Comment #3. The data synthesis included a narrative however, no descriptive data on the quantitative studies is included. Unclear how the thematic analysis from all the studies was used for all the data together.**

We agree this was unclear in our initial submission. We have revised our methods section to clarify this

Consistent with previous systematic reviews, we summarised results and described them narrative. An inductive thematic synthesis was conducted by two authors to analyze the extracted data. This process involved line-by-line coding, theme categorization, and the development of analytical themes, following the approach outlined by Braun and Clarke

**Comment #4. The results need to include actual numbers not mostly or majority.**

We take note and thank the reviwer for the suggestion. We have revised our results to clarify this as per reviewer #1 and reviewer #2 suggestions.

**Fatigability**

Activity tolerance in older adults encompasses the ability to engage in various activities and maintain different body positions, including walking, climbing stairs, and performing kitchen or cleaning tasks. Older adults commonly attribute difficulties in these activities to self-reported musculoskeletal or cardiopulmonary conditions.[1] Two studies also reported how indoor mobility activities of older adults is limited due to reduced endurance or fatigability, leading to slower walking and gait speeds.[2, 3] Indeed, nearly one in every four older adult (22.9%) were reported to experience fatigue when transferring or moving indoor, with 32.1% reported fatigue especially among those with co-morbidities.[2] In another study by Clemencon and colleagues, the authors that leg muscle mass can be an important parameter to use as it predicts fatigability and therefore physical performance.

As individuals age, the prevalence of chronic diseases rises, with more than half of the population (68.8%) requiring pharmacotherapies.[4] The use of medication was found to impact their mobility as older adults reported experiencing fatigue with these medication use. In particular, there was a 10% increase in the likelihood of reporting indoor mobility-related fatigue with every additional medication taken by an older adult.[2] Additionally, older adults reporting musculoskeletal pain were twice as likely to report fatigue, and the presence of depressive symptoms was significantly associated with reported fatigability, which led to reduced mobility.[2] Among frail older adults, this fatigue led to decreased step counts, increased sedentary behaviour, and greater levels of disability compared to their more robust cohort.[4]

**Poor balance**

Functional mobility and balance, stemming from previous falls, are common health barriers among older adults, leading to decreased balance, unsteadiness, and concerns about night time bathroom accessibility.[15] While home-dwelling elders generally exhibit better balance compared to institutionalised elderly individuals, approximately one-third of older adults expressed fear of falling, significantly impeding their mobility.[17] May and colleagues established an inverse relationship between balance (measured by mean sway path) and mobility index, indicating that better balance corresponds to higher mobility scores. Conversely, individuals with a fear of falling display lower mobility, slower walking speeds, and higher mean sway path values, indicating poorer balance.[19]While reasons for the poor balance vary, these were identified due to several factors, including the use of medications, the consumption of alcohol, inner ear imbalance as well as certain medical conditions, such as cognitive impairment.[19] Importantly, for an older adult to be mobile out of doors, this required them to have a moderate or rapid gait speed, which are often lacking among those with poor balance.[5]

The review also noted an association between cognitive impairment and the likelihood of misjudging daily activities, especially those living in nursing homes.[6] These results underscore the importance of considering cognitive state when evaluating self-assessed mobility, especially for those living in nursing homes.[6] Mobility limitations negatively impacted daily activities including feeding, bathing, grooming, dressing, continence (bowels and bladder), toilet use, transfer, mobility, and stairs climbing.[6] Studies also reported that due to hearing loss, bowel and bladder issues, and vision impairments contribute to poor balance, thereby increasing the risk of falls.[5]

**Home environmental barriers**

The home environment was identified as the key factor that can either support or hinder the indoor mobility of older adults, often contributing to falls due to poor balance. In particular, older adults described the importance of creating a safe and accessible living environment at home to support indoor mobility among older adults.[1] For instance, hazards such as damaged or broken stairs and railings, poor lighting, loose areas of rugs and obstacles in walkways were reported to cause these older adults to be wary, as they worry about the risk of falling.[1] Due to these factors, older adults were reported to limit their movements indoors which led to reduced mobility indoors and decline in independence.[5]

**Reduced strength and limb weakness**

Both reduced physical strength and limb weaknesses due to ageing among older adults were reported to significantly impact the ability of older adults to move indoors, with a concomitant toll on access to daily activities. For instance, studies have reported that older adults experience a reduced range of motion, which limits their mobility, flexibility, and walking speed.[7] These limitations were more pronounced among those living in retirement communities, as they were reported to engage in fewer daily steps, and exhibit lower activity levels[4, 7, 8] compared to those living independently in the community[9]. This limitation was even more pronounced among those with frailty, where Brustio and colleagues reported that among frail older adults, they were more likely to experience indoor mobility restrictions and higher levels of disability.[4]

**Comment #5. The conclusion would benefit from a stronger emphasis on the implications for policy and practice.**

We agree and thank the reviewer for the suggestion. Our revised conclusion now reads as follow

In summary, this study identified challenges older adults face in indoor mobility, including physical limitations, frailty, chronic diseases, medication use, and environmental barriers. To optimize implementation, future studies should examine the cost-effectiveness of different implementation models across diverse geographic and socioeconomic contexts to create more adaptable approaches to aging-in-place. Our findings also challenge the current paradigm that aging-in-place can succeed without systematic support structures. The evidence demands not just acknowledgment of these challenges but decisive action to transform how we support indoor mobility among older adults.

**Comment #6. The main themes are presented without a general description of the theme and no supporting results such as quote, or actual numbers are presented for the themes.**

We take note and thank the reviewer for pointing this out. We have now revised our results to clarify this. We have also generated an image to enhance the presentation of our themes as follow

Figure 2: Indoor mobility challenges of older adults

**Comment #7. In the discussion some of the ideas discussed are not related to the results presented previously. There is no integration of the qualitative and quantitative results. The discussion is generics. Examples need to consider socioeconomic factors was this data not available or not extracted from the articles.**

We agree. We have now revised our results section as suggested by the reviewer and clarified that there were only one qualitative study and eight quantitative studies. As these studies did not specifically examine the socioeconomic factors on how this can enhance or impede indoor mobility, we are unable to provide an example. However, we have included this as a suggestion for future research as follow:-

**Socioeconomic Factors and Determinants of Indoor Mobility Challenges**

To date, there is limited understanding of how socioeconomic factors affect indoor mobility among older adults. As such further exploration and understanding how socioeconomic status (e.g., income, education, and housing affordability) affects indoor mobility will help identify specific barriers and facilitate the development of strategies that promote equitable access to resources. Understanding how socioeconomic factors shape the physical environment, access to assistive devices, social support networks, caregiving arrangements, and healthcare utilisation is vital for developing interventions that address the underlying determinants of indoor mobility challenges. From the perspective of the Selection, Optimization, and Compensation (SOC) model[13] of successful ageing, older adults who set goals to maximise their mobility capabilities are more likely to experience a better quality of life. Implementing multiple strategies to address mobility challenges, such as optimising available resources and compensating for physical limitations, could empower older adults to maintain their independence and improve their overall well-being.

**Comment #8. Conceptually it is unclear how long-term care institutions and community houses were grouped together when the cohort of older people is different. Also, long-term care institutions are usually licences and have minimum environmental standards to people who are frail while personal houses do not. Presenting the data jointly limits the analysis and possible recommendations.**

We take note of the suggestion. We have now included an additional section on this as an area for future research

**Living environment**

Our review also suggests that there may be baseline differences in terms of frailty and cognitive ability of older adults living in the community and institutional settings.[5, 7] As such, different levels of interventions may need to be tailored for these individuals from different setting. For instance, as older adults living in institutional settings are more likely to be frail, they may require interventions focusing on compensatory strategies compared to those living in the community which may require exercises to improve their strength and balance. Future research should explicitly separate and compare indoor mobility challenges between institutional and community settings to develop more targeted and supportive care approaches.

We trust that these changes will meet the requirements of the journal and the reviewer. We look forward to contributing to your esteemed journal

Yours truly

Shaun LEE on behalf of the authors
