# Supplementary material for: Indoor mobility challenges among older adults: A systematic review of barriers and limitations
Source: PLoS One. 2025 Jun 4;20(6):e0325064. doi: 10.1371/journal.pone.0325064 (PMC12136408; doi:10.1371/journal.pone.0325064)
Supplement: Attachment — Submitted filename: Cover letter.docx [file pone.0325064.s009.docx]

16 April 2025

Jan Chrusciel

Academic Editor

PLOS ONE

Dear Dr Jan Chrusciel,

Thank you very much for kindly considering our manuscript entitled “Indoor mobility challenges among older adults: A systematic review of barriers and limitations” for submission to PLOS ONE. As requested by the reviewer, we have made substantial changes to our manuscript. These changes are delineated as follow:-

**Reviewer #1 had the following comment:-**

Comment#1: Thank you for your revisions and for addressing the comments from the previous review. Below are my observations and suggestions regarding the revised manuscript:

1. Adequacy of Revisions
The manuscript has improved significantly, and most of the previous concerns have been addressed and this manuscript is now acceptable for publication

2. Technical Soundness & Data Support for Conclusions
The study is technically sound, and the methodology aligns with the research objectives.

The conclusions are generally supported by the data.

3. Statistical Analysis
The statistical methods is not applicable for this study.


4. Data Availability & Transparency
The authors have provided the necessary data to support their findings.

5. Additional Comments
NA

Overall, the manuscript is well-structured and contributes meaningfully to the field. With minor adjustments, it will be suitable for publication

Thank you very much for the encouraging comments. We look forward to contributing to the esteemed journal.

**Reviewer #2 had the following 2 minor comments**

Comment #1: Thank you for addressing all the raised comments. The title includes barriers but this was not included within the search strategy.

We agree this was unclear. In our initial submission, we had only used the challenges in our title but this was changed based on the suggestion of the reviewer. Irrespective of this, our search strategy started with a broad search of the literature to which our studies screened and only which included those on barriers and issues on mobility. This is described in our methods section, under study eligibility criteria as below:-

## **Study eligibility criteria**

We included studies of any design (e.g. cohort, cross-sectional, case reports, qualitative) that examined the indoor space environment and mobility challenges experienced by an older adult aged 60 years and above, published in English language.

Comment #2. It is unclear how reflexive thematic analysis by Braun and Clark was used on the included studies when these were cross-sectional using varied questionnaires or physical measures.

We agree this was unclear. To clarify this, we added a statement to describe our method where we had first read and understood the meaning and patterns related to the physical measures and themed them subsequently. The revised method reads as follow:-

Data from various questionnaires were also themed by analysing the underlying meaning and patters within the numerical data.

We trust that these changes will meet the requirements of the journal and the reviewer. We look forward to contributing to your esteemed journal

Yours truly

Shaun LEE on behalf of the authors
